# The laccase-like reactivity of manganese oxide nanomaterials for pollutant conversion: rate analysis and cyclic voltammetry

**DOI:** 10.1038/s41598-017-07913-2

**Published:** 2017-08-10

**Authors:** Xinghao Wang, Jiaoqin Liu, Ruijuan Qu, Zunyao Wang, Qingguo Huang

**Affiliations:** 1 State Key Laboratory of Pollution Control and Resources Reuses, School of the Environment, Nanjing University, Nanjing, 210023 P. R. China; 2 College of Agricultural and Environmental Sciences, Department of Crop and Soil Sciences, University of Georgia, Griffin, Georgia 30223 United States

## Abstract

Nanostructured manganese oxides, e.g. MnO_2_, have shown laccase-like catalytic activities, and are thus promising for pollutant oxidation in wastewater treatment. We have systematically compared the laccase-like reactivity of manganese oxide nanomaterials of different crystallinity, including α-, β-, γ-, δ-, and ɛ-MnO_2_, and Mn_3_O_4_, with 2,2′-azinobis-(3-ethylbenzthiazoline-6-sulfonate) (ABTS) and 17β-estradiol (E2) as the probing substrates. The reaction rate behaviors were examined with regard to substrate oxidation and oxygen reduction to evaluate the laccase-like catalysis of the materials, among which γ-MnO_2_ exhibits the best performance. Cyclic voltammetry (CV) was employed to assess the six MnO_x_ nanomaterials, and the results correlate well with their laccase-like catalytic activities. The findings help understand the mechanisms of and the factors controlling the laccase-like reactivity of different manganese oxides nanomaterials, and provide a basis for future design and application of MnO_x_-based catalysts.

## Introduction

Enzyme-catalyzed oxidative reactions are increasingly examined as an alternative approach to water/wastewater treatment and soil remediation for decomposing organic pollutants, but are limited by enzyme denaturation and cost. Extensive efforts have been made to develop man-made materials that can be used to mimic the catalytic function of natural enzymes, also known as artificial enzymes. In particular, a variety of nanomaterials have been explored for potential use as artificial enzymes^1, 2^, which have also been referenced as nanozymes^3, 4^.

Manganese oxides nanomaterials have recently been found to exhibit reactivity similar to laccase, a phenol oxidase that has promising application in pollution control, and have been named “nanozymes” in recent studies^5–7^. Such naming may however be premature, given that no comprehensive information is available regarding the laccase-like activity of manganese oxides nanomaterials. There are more than thirty different naturally occurring crystal forms of manganese oxides (MnO_x_), which are major components of soils and sediments, participating in a variety of natural chemical reactions^8, 9^. A number of previous studies have only investigated the oxidative reactivity of MnO_x_ towards organic pollutants, and have shown that the crystallinity of the materials plays an important role controlling the reactivity^10–14^. There has however been little progress in understanding the mechanisms responsible for the laccase-like activity of manganese oxides, which is the aim of this study.

**Figure 1:**
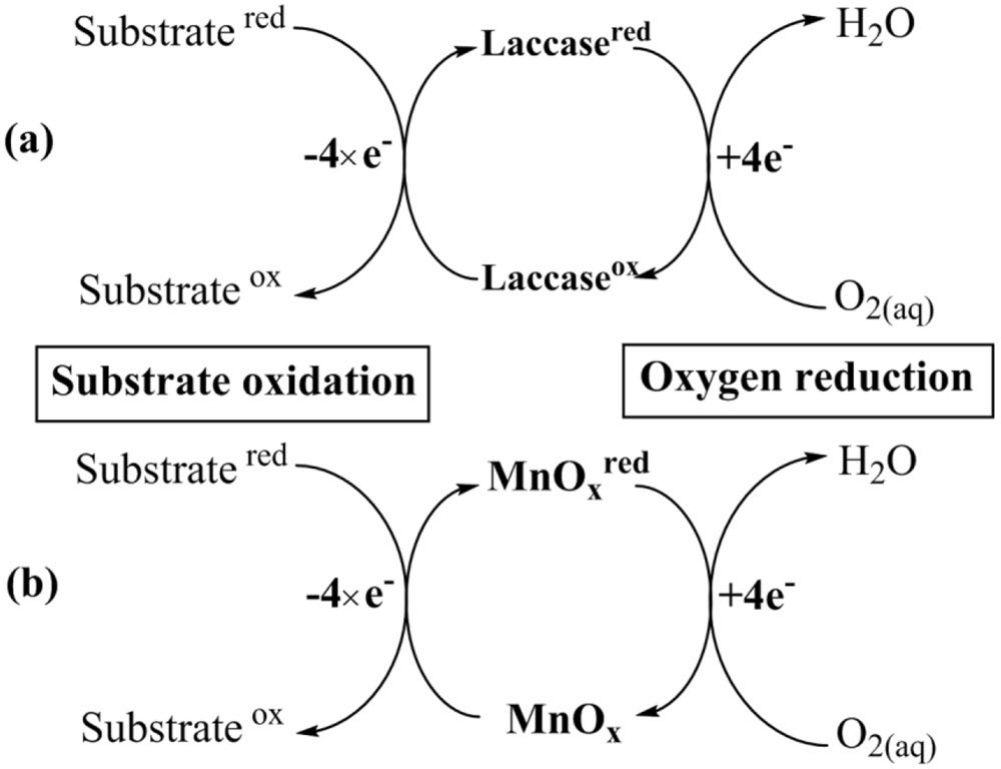
A conceptual model of the laccase-like reactions of MnO_x_ nanomaterials.

It is well known that the copper-cluster enzyme laccase catalyzes one-electron oxidative reaction of substrates, while molecular oxygen undergoes four-electron reduction to water as shown in Fig. 1 (part a), in which the copper redox reactivity plays a critical role in shuttling the electrons from substrates to oxygen^15, 16^. The substrates of laccase include many organic contaminants, which comprises the basis for laccase use in environmental applications. Some substrates change color upon laccase oxidation, such as 2,2′-azinobis-(3-ethylbenzthiazoline-6-sulfonate) (ABTS), which have been employed in methods to assess laccase activity. Similar to laccase, certain manganese oxides (MnO_x_) can also oxidize substrates via single electron transfer^10, 11, 17, 18^, while the resultant reduced manganese oxides MnO_x_^red^ can be re-oxidized to MnO_x_ by dissolved oxygen that is reduced to water under certain conditions^19–21^ (Fig. 1 part b), leading to a net result of electron shuttling from substrates to oxygen, like laccase. In addition, manganese oxides and laccase share some common substrates, including organic contaminants and ABTS, which provides manganese oxides laccase-like reactive capacity. As a result, manganese oxide nanomaterials have been suggested to be possible cost-effective catalysts for wastewater treatment^22^.

It should be noted that a manganese oxide, or its nano-sized form, could not be regarded as a catalyst, unless it can undergo both the substrate oxidation and the oxygen reduction reactions illustrated in Fig. 1(b), so that MnO_x_ can complete a full redox cycle to return to its oxidation state. It is therefore premature to describe manganese oxide nanoparticles as nanozymes, simply because they can oxidize ABTS to change color, as does in laccase activity assessment. It should also be noted that the catalytic performance of a manganese oxide is dependent on both sides of the redox cycle shown in Fig. 1(b), i.e. the substrate oxidation and the oxygen reduction, and the overall rate is limited by the slower side.

This study systematically investigated the laccase-like reaction behaviors of six manganese oxide nanomaterials of different crystallinity by assessing their reactivity towards two model substrates, ABTS and 17β-estradiol (E2), and the reaction rates of both substrate oxidation and oxygen reduction were evaluated. Cyclic voltammetry (CV) was employed to assess the six MnO_x_ nanomaterials, in an attempt to explore the mechanisms of and the factors controlling their laccase-like reactivity. Although CV is a powerful tool to examine electrochemical property and reactivity^23, 24^, the work to use CV on the laccase-like reactions of different crystalline manganese oxides has been rather limited.

## Results and Discussion

**Figure 2:**
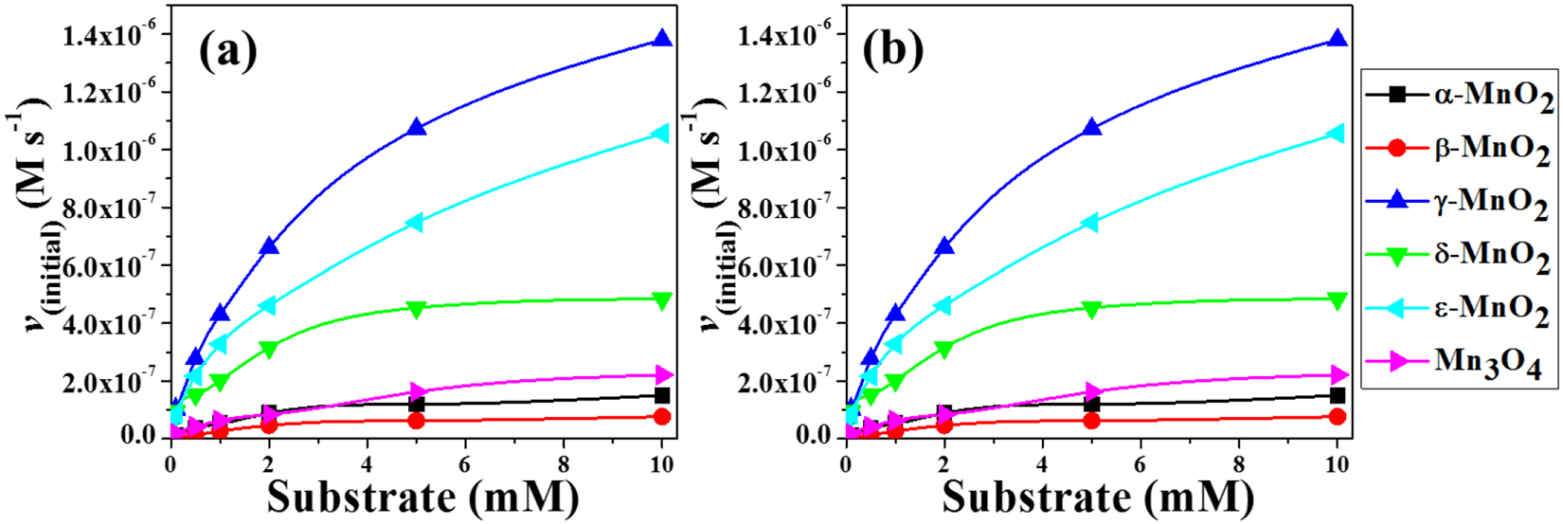
(**a**) The initial reaction rate of ABTS (0.01, 0.1, 0.5, 1, 2, 5, and 10 mM) by six MnO_x_ nanomaterials (336 μM) at pH 6.0, and (**b**) the double-reciprocal plots.

The initial reaction rate of ABTS oxidation by each of the six synthesized manganese oxide nanomaterials (α-, β-, γ-, δ-, and ε-MnO_2_ and Mn_3_O_4_) was investigated spectrophotometrically at room temperature for the first 5 minutes (Fig. 2). The product of ABTS oxidation has a characteristic absorbance at 420 nm, and the increase of this absorbance over time is evident resulting from ABTS oxidation by MnO_x_ (Fig. S3), of which the ABTS reaction rates by γ-MnO_2_ and ε-MnO_2_ are indeed greater than the natural laccase at the same dosage (5 mg/mL). The concentration of ABTS oxidation product can be calculated by Bill’s law (ε_420_ = 36 000 M^−1^ cm^−1^)^25^, and the rate of its formation over the first 5 minutes was used to evaluate the initial reaction rate (*v*_initial_), that is the slope of the linear fitting (Fig. S3). The initial reaction rate measured with different substrate concentration (0.01, 0.1, 0.5, 1.0, 2.0, 5.0, and 10 mM of ABTS) was fitted to Lineweaver-Burk equation 1/*v*_initial_ = K_m_/*v*_m_ (1/[S] + 1/K_m_), where K_m_ is the Michaelis-Menten constant, *v*_m_ is the maximum reaction rate, and [S] is the substrate concentration. Further, *k*_cat_ can be calculated from dividing *v*_m_ by the MnO_x_ concentration. The results of data fitting for all six MnO_x_ are listed in Table 1, and the Lineweaver-Burk double-reciprocal plots are displayed in Fig. 2b.

**Table 1:** Rate constants for the laccase-like reactions of different manganese oxide nanomaterials with ABTS as the substrate.

Types	pH	*K*_m_ (mM)	*v*_max_ (M s^−1^)	*k*_cat_/*K*_m_ (M^−1^ s^−1^)	*k*_cat_ (s^−1^)	*k*_O2_ (M s^−1^)	*k*_3_ (M^−1^ s^−1^)	*k*_5_ (M^−1/4^ s^−1^)	*k*_Mn(II)_ (M s^−1^)
α-MnO_2_	6.0	1.97	1.68 × 10^−7^	0.2534	4.99 × 10^−4^	9.86 × 10^−11^	503	4.58 × 10^−6^	1.34 × 10^−8^
β-MnO_2_	6.0	4.54	1.34 × 10^−7^	0.0876	3.98 × 10^−4^	4.89 × 10^−11^	401	4.22 × 10^−6^	7.24 × 10^−8^
γ-MnO_2_	6.0	2.25	1.49 × 10^−6^	1.9648	4.42 × 10^−3^	1.82 × 10^−10^	4452	4.71 × 10^−6^	2.42 × 10^−7^
δ-MnO_2_	6.0	1.24	5.14 × 10^−7^	1.2313	1.53 × 10^−3^	1.48 × 10^−11^	1542	4.52 × 10^−7^	2.05 × 10^-7^
ε-MnO_2_	6.0	1.86	9.98 × 10^−7^	1.5937	2.97 × 10^−3^	6.42 × 10^−11^	2991	1.85 × 10^−6^	2.17 × 10^−7^
Mn_3_O_4_	6.0	2.22	2.19 × 10^−7^	0.2922	6.50 × 10^−4^	3.42 × 10^−11^	655	1.15 × 10^−6^	1.86 × 10^−7^

As shown in Table 1, the maximum reaction rate (*v*_m_) varies from 1.34 × 10^−7^ to 1.49 × 10^−6^ M s^−1^ for the six MnO_x_ nano materials at 336 μM, with γ-MnO_2_ being the highest, while the K_m_ ranges from 1.86 mM to 4.54 mM. In terms of *k*_cat_/K_m_, the laccase-like oxidative performances of the six nanomaterials decrease in the order: γ-MnO_2_ > ε-MnO_2_ > δ-MnO_2_ > Mn_3_O_4_ > α-MnO_2_ > β-MnO_2_. The difference in reactivity of the six MnO_x_ nanomaterials may arise from their different crystal structures^26^.

**Figure 3:**
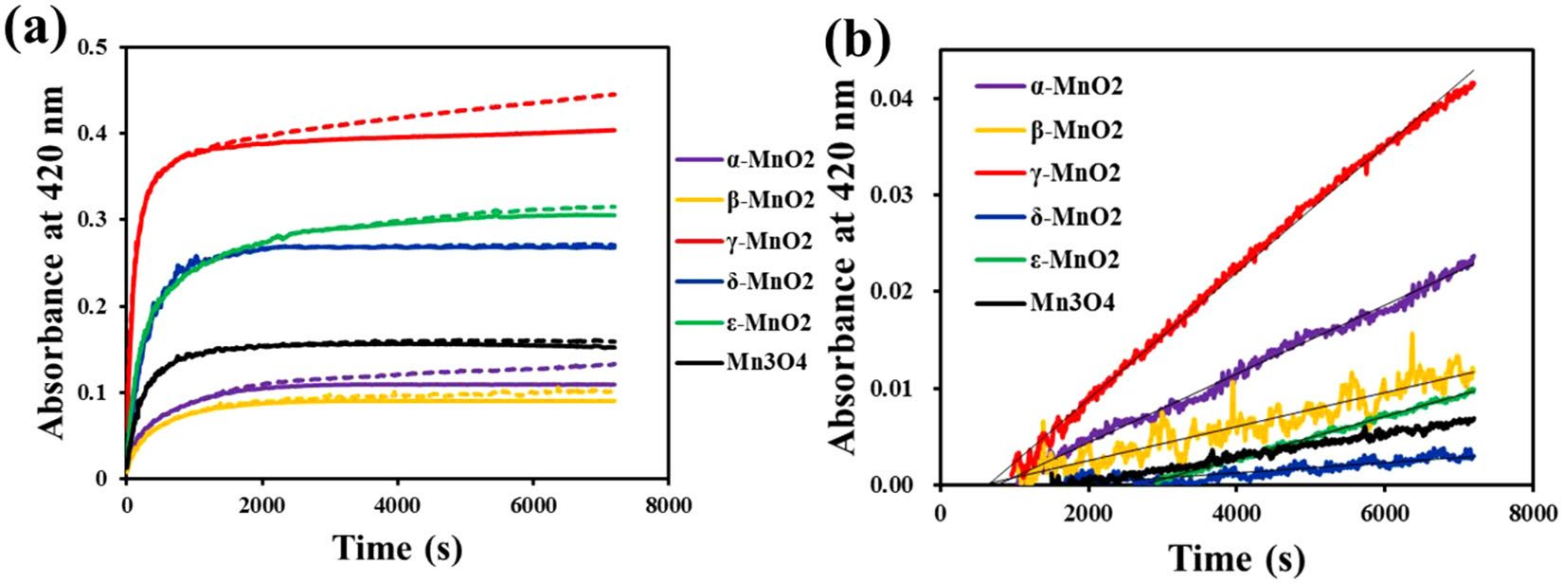
(**a**) The absorbance change from ABTS (2 mM) oxidation by MnO_x_ (10.0 μM) at pH 6.0 in systems with O_2_ saturated (21.5 mg/L, dash line) or depleted (0.30 mg/L, solid line); (**b**) The difference in absorbance between the two systems tested in a for each MnO_x_.

In order to explore the role of oxygen in the laccase-like reactivity of different manganese oxides, ABTS oxidation was monitored in systems with O_2_ saturated (21.5 mg/L) or depleted (0.30 mg/L) (Fig. 3). It is interesting that ABTS oxidation by MnO_x_ did not exhibit difference between the two systems initially as seen in Fig. 3a, but the difference became evident after about 15 minutes of reaction when the absorbance of ABTS oxidation product at 420 nm became plateaued in the oxygen-depleted system, while that in the oxygen-saturated systems kept increasing steadily, albeit at a rate slower than the initial reaction stage.

As schematically represented in Fig. 1, the laccase-like reactivity of MnO_x_ is composed of two reactions that form a catalytic cycle. On one side of the cycle (Fig. 1), the substrate is oxidized, while MnO_x_ is reduced to MnO_x_^red^ (i.e. MnO_2_ to MnOOH and MnOOH to Mn(II)); and on the other side, MnO_x_^red^ is oxidized by oxygen (e.g. Mn(II) to MnOOH). The result in Fig. 3a suggests that MnO_x_^red^ oxidation by oxygen is much slower than MnO_x_ reduction, and thus, during the initial reaction stage, ABTS oxidation did not exhibit difference between the systems with or without oxygen; but when MnO_x_ was fully reduced at the later stage, the ABTS oxidation stopped in the oxygen-depleted system, while kept going steadily in the oxygenated system where Mn^2+^ was oxidized back to MnOOH by oxygen. The difference between the two systems in absorbance increased linearly over time for all six MnO_x_ nanoparticles (Fig. 3b), from which the overproduction of ABTS oxidation product (ε_420_ = 36 000 M^−1^ cm^−1^) in the oxygenated system can be calculated, which was found to fit zero-order rate equation, and the rate constants (*k*_O2_) thus obtained (M s^−1^) are listed in Table 1. In terms of *k*_O2_, the contribution of oxygen on the ABTS oxidation by MnO_x_ followed the order of γ-MnO_2_ > α-MnO_2_ > ε-MnO_2_ > β-MnO_2_ > Mn_3_O_4_ > δ-MnO_2_.

A rate analysis on the catalytic cycle of MnO_x_ presented in Fig. 1 is given below, using MnO_2_ as the model catalyst. The substrate oxidation can be represented in three steps, as it is a heterogeneous reaction. The substrate first adsorbs onto MnO_2_ (equation 1), followed by oxidation by MnO_2_ which was transformed to its reduced form MnOOH (equation 2), and then to aqueous Mn(II) (equation 3), and it is known that MnOOH is unstable and the reaction from MnOOH to Mn(II) is fast in the presence of substrate^27, 28^. In the presence of oxygen, Mn(II) can be oxidized to MnOOH (equation 4)^19^, which appears to be rate limiting in the long run as discussed above.

1 $${{\rm{MnO}}}_{{\rm{2}}}+{\rm{S}}\underset{{k}_{2}}{\overset{{k}_{1}}{\rightleftharpoons }}{{\rm{MnO}}}_{{\rm{2}}}\mathrm{...}{\rm{S}}$$

2 $${{\rm{MnO}}}_{{\rm{2}}}\mathrm{...}{\rm{S}}+{{\rm{H}}}^{+}\,\mathop{\longrightarrow }\limits^{{k}_{3}}\,{\rm{MnOOH}}+{\rm{P}}$$

3 $${\rm{MnOOH}}+{{\rm{3H}}}^{+}+{\rm{S}}\,\mathop{\longrightarrow }\limits^{{k}_{4}}\,\mathrm{Mn}(\mathrm{II})+{{\rm{2H}}}_{{\rm{2}}}{\rm{O}}+{\rm{P}}$$

4 $$\mathrm{Mn}(\mathrm{II})+{1/\mathrm{4O}}_{{\rm{2}}}+{3/\mathrm{2H}}_{{\rm{2}}}{\rm{O}}\,\mathop{\longrightarrow }\limits^{{k}_{5}}\,{\rm{MnOOH}}+{{\rm{2H}}}^{+}$$

Two specific situations are considered here. The first is when the reaction is at its initial stage, and the consumption of MnO_2_ is relatively minor, so that $[{{\rm{MnO}}}_{2}]\approx {[{{\rm{MnO}}}_{2}]}_{0}-[{{\rm{MnO}}}_{2}{\rm{\ldots }}{\rm{S}}]$.

A pseudo-steady state was approached to [MnO_2_…S] in equation 5, which was rearranged into equation 6, given $[{{\rm{MnO}}}_{2}]\approx {[{{\rm{MnO}}}_{2}]}_{0}-[{{\rm{MnO}}}_{2}{\rm{\ldots }}{\rm{S}}]$.

5 $$\frac{{\rm{d}}[{{\rm{MnO}}}_{2}{\rm{\ldots }}{\rm{S}}]}{{\rm{dt}}}={k}_{1}[{{\rm{MnO}}}_{2}]\,[{\rm{S}}]-{k}_{2}[{{\rm{MnO}}}_{2}{\rm{\ldots }}{\rm{S}}]-{k}_{3}[{{\rm{MnO}}}_{2}{\rm{\ldots }}{\rm{S}}][{{\rm{H}}}^{+}]={\rm{0}}$$

6 $$[{{\rm{MnO}}}_{2}{\rm{\ldots }}{\rm{S}}]=\frac{{k}_{1}{[{{\rm{MnO}}}_{2}]}_{0}\,[{\rm{S}}]}{{k}_{2}+{k}_{3}\,[{{\rm{H}}}^{+}]+{k}_{1}[{\rm{S}}]}\,$$

Based on chemical equations 2 and 3, the rate of oxidation product formation can be written in equation 7,

7 $$\frac{{\rm{dP}}}{{\rm{dt}}}={k}_{3}[{{\rm{MnO}}}_{2}{\rm{\ldots }}{\rm{S}}][{{\rm{H}}}^{+}]+{k}_{4}[{\rm{MnOOH}}]{[{{\rm{H}}}^{+}]}^{3}[{\rm{S}}]$$

Substitution of equation 6 into equation 7 yields equation 8, given $[{\rm{MnOOH}}]\approx {\rm{0}}$ in the initial reaction stage.

8 $$\frac{{\rm{dP}}}{{\rm{dt}}}=\frac{{k}_{3}{[{{\rm{MnO}}}_{2}]}_{0}{[{\rm{H}}}^{+}][{\rm{S}}]}{\frac{{k}_{2}+\,{k}_{3}[{{\rm{H}}}^{+}]}{{k}_{1}}+[{\rm{S}}]}=\frac{{{\rm{v}}}_{{\rm{\max }}}[{\rm{S}}]}{{K}_{{\rm{m}}}+[{\rm{S}}]}$$

It is noted that equation 8 takes the form of Michaelis–Menten equation, where ${K}_{{\rm{m}}}=\frac{{k}_{2}+\,{k}_{3}[{{\rm{H}}}^{+}]}{{k}_{1}}$, and ${v}_{{\rm{\max }}}=\,{k}_{3}{[{{\rm{MnO}}}_{2}]}_{0}[{{\rm{H}}}^{+}]$. This is consistent with the results expressed in Fig. 2 and Table 1, where *k*_cat_ = *k*_3_[H^+^], based on which *k*_*3*_ can be calculated and is also listed in Table 1.

The second situation that we have considered is when MnO_2_ was exhausted, so that [Mn(II)] reached pseudo-steady state.

A pseudo-steady state was approached to [Mn (II)] based on chemical equations 3 and 4.

9 $$\frac{{\rm{d}}[\mathrm{Mn}(\mathrm{II})]}{{\rm{dt}}}={k}_{4}[{\rm{MnOOH}}]{[{{\rm{H}}}^{+}]}^{3}[{\rm{S}}]-{k}_{5}[\mathrm{Mn}(\mathrm{II})]{[{{\rm{O}}}_{2}]}^{\frac{1}{4}}=0$$

Based on chemical equation 3, the rate of oxidation product formation can be written in equation 10, to which equation 9 is combined.

10 $$\frac{{\rm{d}}[{\rm{P}}]}{{\rm{dt}}}={k}_{4}[{\rm{MnOOH}}]{[{{\rm{H}}}^{+}]}^{3}[{\rm{S}}]={k}_{5}[\mathrm{Mn}(\mathrm{II})]{[{{\rm{O}}}_{2}]}^{\frac{1}{4}}$$

It is noted that equation 10 is a zero-order rate equation, consistent with the result presented in Fig. 3 and Table 1, where $\,{k}_{{{\rm{O}}}_{{\rm{2}}}}={k}_{5}[{\rm{Mn}}({\rm{II}})]{[{{\rm{O}}}_{2}]}^{\frac{1}{4}}$.

The rate equations (8) and (10) are in good accord with the rate data collected respectively at the initial reaction stage when MnO_2_ is fresh and the later stage when MnO_2_ is exhausted. The rate analysis reveals that the substrate is oxidized by MnO_2_ at the initial stage, which rate can be well modeled by Michaelis–Menten equation (equation 8); while, at the later stage, substrate oxidation is limited by the rate of Mn(II) oxidation back to MnOOH, and follows a zero-order equation (equation 10). It should be noted that MnO_2_ behaves essentially as an oxidant (electron acceptor), rather than a catalyst, in the initial reaction stage, as the conversion of Mn(II) back to MnOOH is slow, so that the presence of oxygen has little contribution to the substrate oxidation rate in the initial stage. It is till the late stage of the reaction when MnO_2_ reactivity is exhausted, the oxidation of Mn(II) back to MnOOH starts to control the rate of substrate oxidation, so that the complete catalytic cycle shown in Fig. 1 reaches a steady state, in which MnO_2_ behaves as a catalyst and O_2_ becomes the dominant oxidant. Such an understanding is important when assessing the role of MnO_2_ in environmental reactions, and it is generally desirable to use MnO_2_ as a catalyst rather than an oxidant in environmental applications.

**Figure 4:**
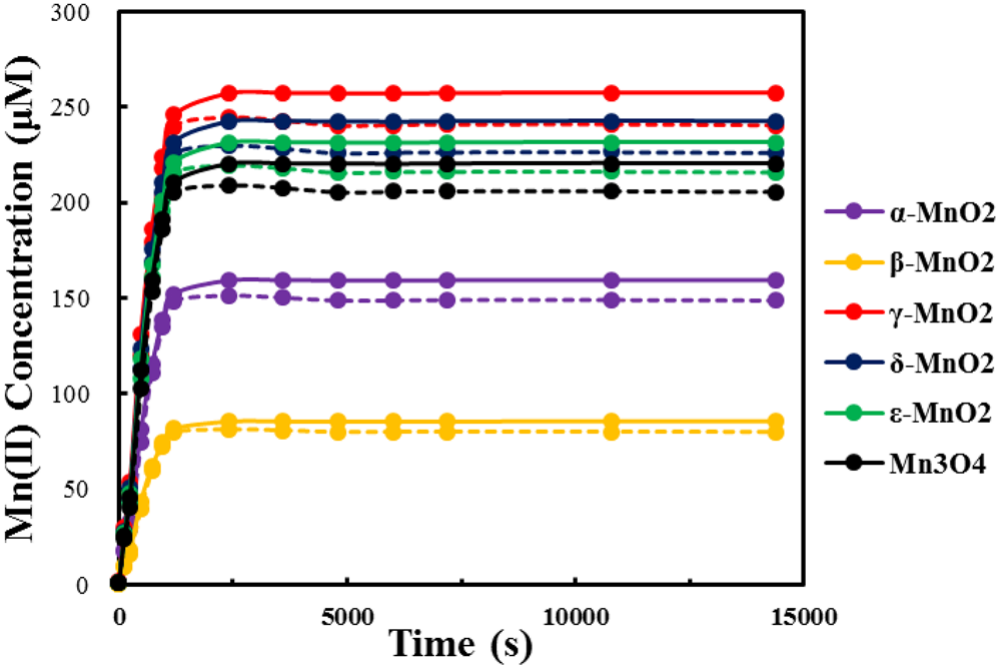
The release of Mn(II) in the laccase-mimicking reactions of ABTS (2 mM) oxidation by MnO_x_ (333.6 μM) at pH 6.0 in systems with O_2_ saturated (21.5 mg/L, dash line) or depleted (0.30 mg/L, solid line).

The concentration of Mn(II) released to aqueous solution during laccase-like reactions was also monitored in systems with O_2_ saturated (21.5 mg/L) or depleted (0.30 mg/L) for different manganese oxides, and the results are presented in Fig. 4. In the initial reaction stage (<900 seconds), the Mn(II) concentration appeared to increase linearly at rates without evident difference between the oxygen saturated and depleted systems, which corroborates the notion that MnO_x_ reduction leads to substrate oxidation during this stage. A rate constant of Mn(II) release (*k*_Mn(II)_) for the initial reaction stage can be calculated according to pseudo-zero order rate equation (Table 1). The concentrations of Mn(II) reached pseudo-steady state at the later reaction stage (>1200 seconds), confirming the assumptions used in the derivation of equation 9 above. The Mn(II) levels were quite different for different MnO_x_ materials, all of which were below the initial MnO_x_ dosage (333.6 µM). This indicates that not all MnO_x_ participated in reaction, probably due to passivation that are dependent on the reaction rate, specific surface area and crystallinity. For each MnO_x_ material, the Mn(II) level in the oxygen-saturated system is below the oxygen-depleted one, but only slightly. The difference should be caused by the Mn(II) oxidation to MnOOH (chemical equation 4) that has supported the continuing oxidation of substrates, but such a process is slow. Therefore, further improvement of the laccase-like reactivity of MnO_x_ shown in Fig. 1 is hinged on improving the oxidation rate of Mn(II). Based on the equatio ${k}_{{{\rm{O}}}_{2}}=\,{k}_{5}[\mathrm{Mn}(\mathrm{II})]{[{{\rm{O}}}_{2}]}^{\frac{1}{4}}$ derived above, the rate constant *k*_5_ can be calculated with the measured Mn(II) concentrations in the oxygen-saturated systems, and listed in Table 1.

**Figure 5:**
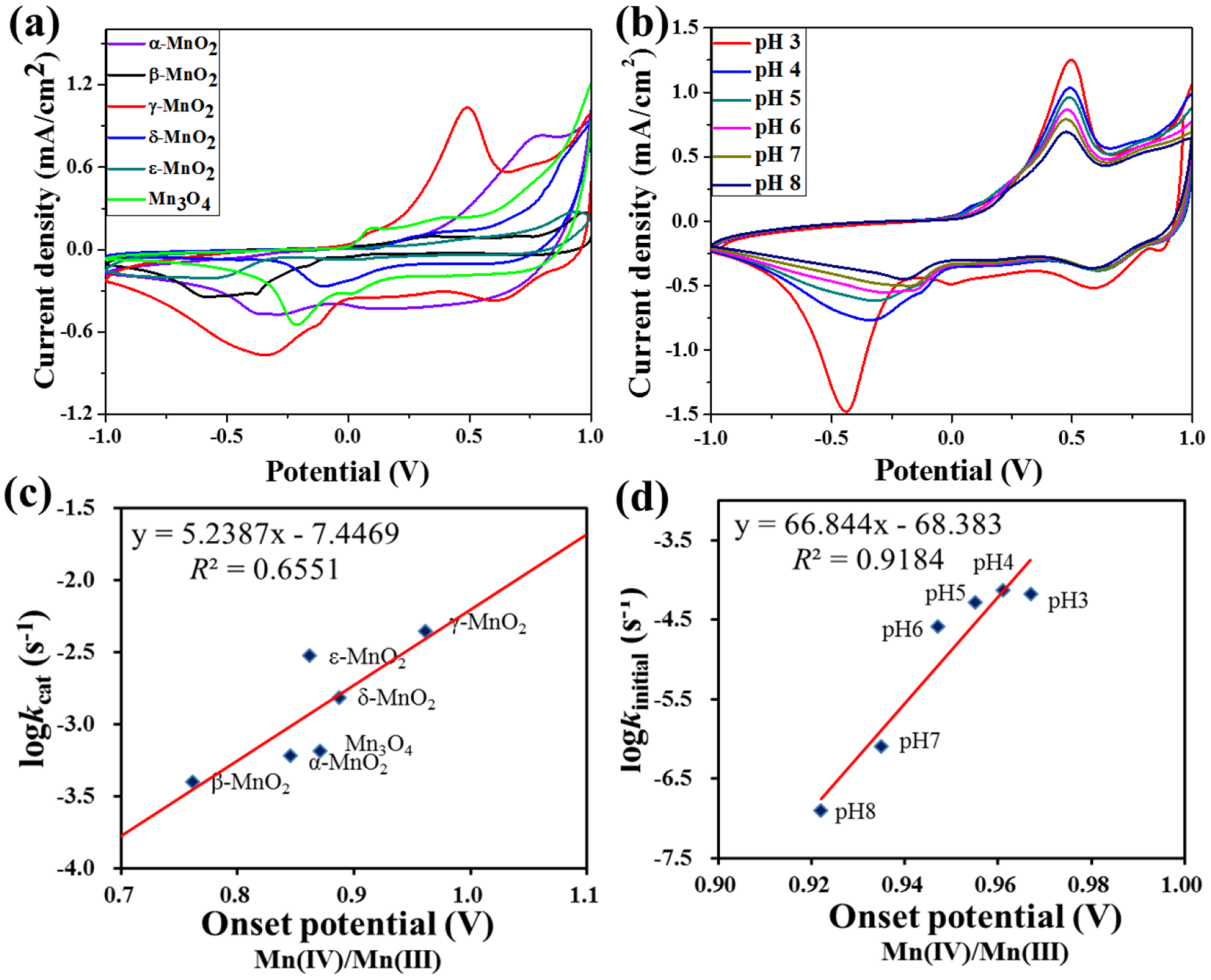
Cyclic voltammetry (CV) at the rate of 20 mV/s in acetate buffer containing 1.0 M KCl and no O_2_ for (**a**) different MnO_x_ at pH 6.0, and (**b**) γ-MnO_2_ at different pH levels. The correlation between the reaction rate constants of the initial ABTS reactions and the onset potential of Mn(IV)/Mn(III) for (**c**) different MnO_x_ at pH 6.0, and (**d**) γ-MnO_2_ at different pH levels.

Cyclic and linear sweep voltammetry was collected for each of the six MnO_x_ nanomaterials to explore the factors controlling their redox reactivity. To this end, MnO_x_ nanoparticles were immobilized on the surface of glassy carbon electrodes that were tested in a three-electrode system. Figure 5a shows the cyclic voltammograms of all six manganese oxides in 1.0 M KCl electrolyte at pH 6.0. Three reduction peaks can be discerned for MnO_2_ nanoparticles; taking γ-MnO_2_ for example, the three peaks are at +0.63, −0.12, and −0.32 V (vs Ag/AgCl), respectively. The first, second and third negative-going reduction peaks can be attributed to the reduction of γ-Mn(IV)O_2_ to manganese oxyhydroxide Mn(III)OOH, Mn(III)OOH to Mn(II) and Mn(IV)O_2_ to Mn(II), respectively^29–31^. In the positive-going scan, three oxidation peaks can also be seen for MnO_2_ materials; taking γ-MnO_2_ for example, the three peaks are located at +0.15, +0.47 and +0.81 V, respectively, corresponding to the oxidation of Mn(II) to MnO_2_, Mn(III)OOH to MnO_2_, and Mn (II) to MnO_4_^−^, respectively. These reduction and oxidation potentials are close to those reported in earlier studies^32–34^.

It is noticeable that the reduction of γ-MnO_2_ to MnOOH begins at +0.83 V, also known as the onset potential. The onset potential reflects the interfacial interaction between the substrate and the electrode surface^35^, including mass transfer and electron transfer, and therefore can be an indicator of the kinetic behavior. It is noted that the onset potential is a quantity measured dependent on the interfacial surface area, and is thus correspondent to the overall rate constant, rather than the one normalized to the surface area. As shown in Fig. 5a, the onset potential of γ-MnO_2_ reduction to MnOOH is the highest among all six MnO_x_ materials, indicating that γ-MnO_2_ reduces most easily, which is consistent with the highest initial reaction rate of ABTS oxidation by γ-MnO_2_ among all six MnO_x_ materials (Table 1). Indeed, the onset potential of Mn(IV)/Mn(III) is correlated to the logarithm of *k*_cat_ of the initial reaction for different MnO_x_ nanoparticles with reasonable goodness of fit (Fig. 5c).

It is noted in equation 7 that pH would play an important role in the initial reaction rate. The initial ABTS oxidation by γ-MnO_2_ nanopaticles under different pH values was also examined, which indicated that γ-MnO_2_ has an excellent oxidation activity at acidic pH values (3.0 and 4.0) than the higher pH values (7.0 and 8.0) (data shown in Fig. S4 and Table S2). The cyclic voltammetry of γ-MnO_2_ has been collected under different pH levels (Fig. 5b). A shift of the reduction peaks towards lower potentials and higher current densities is evident, indicating greater oxidative power of the MnO_x_ materials at more acidic condition. The onset potential of Mn(IV)/Mn(III) is correlated to the logarithm of the initial reaction rate constant (*k*_initial_) of γ-MnO_2_ at different pH (Fig. 5d), which correlation coefficient (*R* ^*2*^ = 0.9184) is much better than that for the different MnO_x_ materials (*R* ^*2*^ = 0.6551, Fig. 5c). The better correlation for γ-MnO_2_ at different pH than for different MnO_x_ materials may suggest that certain factors relating to the crystallinity of MnO_x_ that influence the reactivity may not be captured by the simple relationship between onset reduction potential and the initial reaction rate.

**Figure 6:**
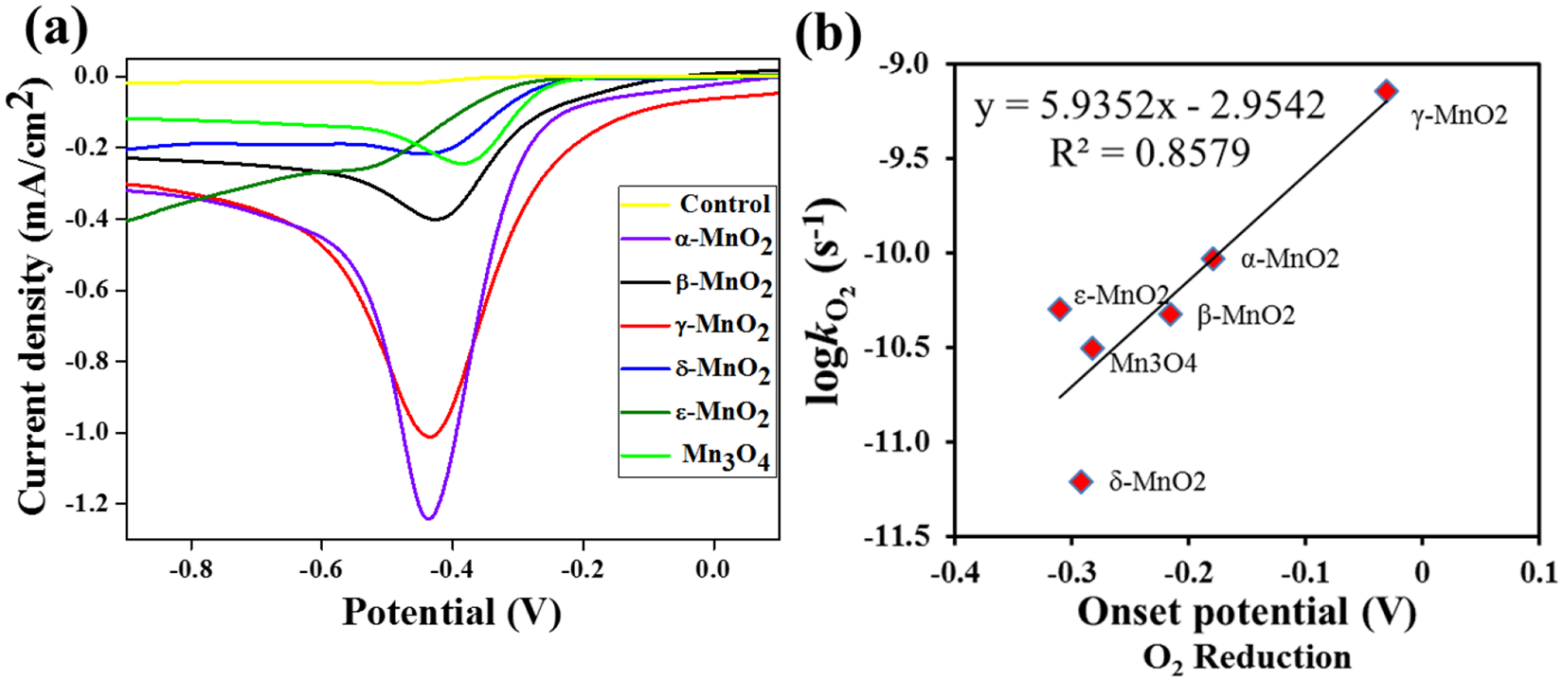
(**a**) Linear sweep voltammetry (scan rate of 20 mV/s) of O_2_ on glassy carbon electrode coated with different MnO_x_ in acetate buffer at pH 6.0 containing 1.0 M KCl. (**b**) The correlation between the log*k*_O2_ of ABTS oxidation and the onset potential of O_2_ reduction with different MnO_x_ nanomaterials.

As indicated in the rate analysis, oxidation of Mn(II) back to MnOOH or oxygen reduction (chemical equation 4) determines the catalytic performance of MnO_x_ in the long run. The reactivity of O_2_ on the six MnO_x_ materials was also probed by linear sweeping voltammetry (Fig. 6). Oxygen reduction peaks are evident, centering around −0.42 V vs Ag/AgCl at pH 6.0, consistent with previous reports^9^. Facilitated oxygen reduction on MnO_x_ is well known, which has been exploited in fuel cells and metal-air batteries^36, 37^. The onset potential of oxygen reduction on MnO_x_ nanoparticles were shifted much higher than that on the glassy carbon electrode without nanoparticles coated, indicating their activity towards oxygen reduction. In terms of the oxygen reduction onset potentials, the oxygen reduction ability follows the order of γ-MnO_2_ > α-MnO_2_ > β-MnO_2_ > Mn_3_O_4_ > δ-MnO_2_ > ε-MnO_2_, which is consistent with the order of their long-term laccase-like zero-order rate constant *k*_O2_ (Table 1), and their linear correlation is present in Fig. 6b. It is reported that the oxygen-manganese clusters enhance the adsorption of oxygen molecules onto MnO_x_ surface and facilitate the electron transfer from the adsorbed oxygen^30, 32^. The Mn-O bond lengths of the six MnO_x_ materials obtained from crystallographic characterization were listed in the Table S3. The most active γ-MnO_2_ possesses the longest Mn-O bond than the other materials. It is possible that the weaker and longer Mn-O in the octahedral (MnO_6_) structure of γ-MnO_2_ is more flexible to facilitate the shuffle of oxygen during oxygen reduction as well as substrate oxidation. Interestingly, the Mn-O bond length exhibits reasonable linear correlation with the *k*_cat_ of the initial reaction and the long-term zero-order rate constant *k*_O2_ (Fig. S5).

**Figure 7:**
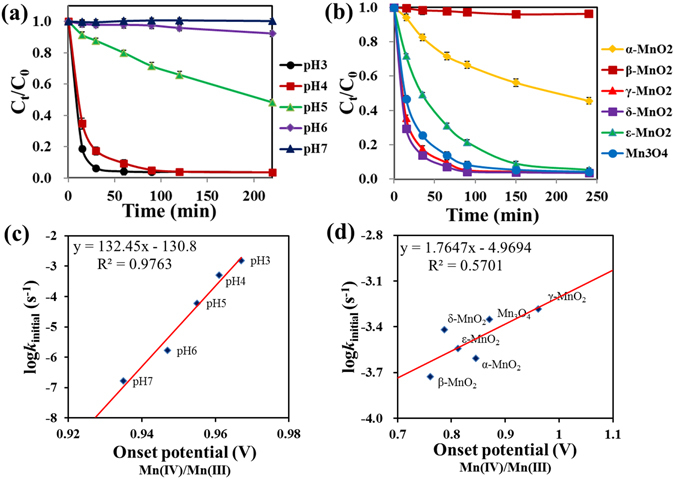
E2 removal in solutions (**a**) containing γ-MnO_2_ under different pH, (**b**) containing different MnO_x_ at pH 4.0. Reaction conditions: initial E2 concentration 10 μM, MnO_x_ dosage 1.0 mM. Error bar represents standard deviations (n = 3). The correlation between log *k*_initial_ of E2 removal and the onset potential of Mn(IV)/Mn(III) for (**c**) γ-MnO_2_ at different pH, and (**d**) different MnO_x_ at pH 4.0.

The initial rate of 17β-estradiol (E2) removal appeared to follow the pseudo-first order kinetics, when γ-MnO_2_ (1 mM) is in excess than E2 (10.0 μM) over the pH range from 3.0 to 7.0 (Fig. 7a), or different MnO_x_ nanoparticles (1 mM) at pH 4.0 (Fig. 7b). The initial reaction rate constant (*k*_initial_) was obtained by fitting the time course E2 concentration to the pseudo-first order rate equation ln(*C*_0_/*C*_t_) = *k*_initial_·t, where *C*_0_ is the initial E2 concentration, and *C*_t_ is the residual E2 concentration at reaction time t. The initial reaction rate constants are linearly correlated to the onset potentials of Mn(IV)/Mn(III) (Fig. 7c and d). We also tested the longer-term reaction rate when E2 (10 µM) is in excess than MnO_x_ (2 µM) in systems at pH 4.0 with O_2_ saturated or depleted (Fig. S6), and a good linear correlation (R^2^ = 0.8187) was found between logarithm of the rate constant *k*_O2_ and the onset potential of O_2_ reduction by different MnO_x_ nanomaterials. The results are consistent with those obtained with ABTS, suggesting the universal applicability of the mechanistic understanding by the rate and voltammetry analysis described above.

According to the removal experiments of 17-β estradiol in water by MnO_x_, the additional TOC measurements have been performed during the reaction at different sampling points. Figure S7 shows the comparison of removal percentage rate of the TOC vs. reaction time in solution with E2_0_ = 10 μM in the presence of six different manganese oxides with 1.0 mM. It can be observed that no obvious removal of TOC occurred in the first half an hour for all nanomaterials, but γ-MnO_2_, δ-MnO_2_, ε-MnO_2_, and Mn_3_O_4_ can efficiently remove 85% more TOC than α-MnO_2_ and β-MnO_2_ after 1 hour reaction. Within the next one hour reaction, the 95.0% of TOC removal was also observed for α-MnO_2_. But for β-MnO_2_, the removal of the TOC can be neglected from Fig. S7, which is consistent with the data of E2 removal (Fig. 7).

The product intermediates of E2 conversion in the presence of γ-MnO_2_ were detected, and two main reaction pathways were proposed involving single-electron coupling and two-electron oxidation shown in Fig. S8. In the single-electron reaction, five peaks with m/z = 541 were identified by the MS analysis (negative ion model) in the early stage of the laccase-like reaction, which likely correspond to the deprotonated ions of the five different E2 dimers (MW = 542) in Fig. S8. It’s because that three types of free radicals were formed in laccase-like reactions by single-electron transfer, and then coupled at different positions via C-C or C-O-C to form five different E2 self-coupling isomer dimers^38, 39^. For the two-electron oxidation, the molecular weights of products (P1, P2, P3, P4, and P5) are larger than that of E2 (MW = 272), suggesting the addition of one oxygen atom to form 2-hydroxyestradiol (P1) is the initial oxidative reaction by MnO_2_. The two hydroxyl group in the aromatic ring of 2-Hydroxyestradiol was then attacked via losing 2 H to form quinone-like product 2, which was further oxidized by inserting an O atom to form product 3. In the next step, a series of aromatic ring-opening products (P4, P5, and P6) and smaller organic molecule were formed, which is consistent with the results from previous studies^40, 41^. The last step of the E2 degradation by MnO_2_ involved complete mineralization of smaller organic molecule to CO_2_ and H_2_O.

According to the above product identification, the initial oxidation reaction of E2 by MnO_2_ can be considered as the single-electron conversion, which is similar to the natural laccase-mediated reaction. But for the long-time reaction, the mineralization of E2 mainly due to the two-electron oxidation, which seems likely that MnO_2_ oxidation can be an effective tool for removing E2 during water and wastewater treatment.

## Conclusion

The nanostructured manganese oxides with different crystallinity have shown laccase-like catalytic activities for pollutant oxidation in wastewater treatment. This study also provides a systematic assessment of the voltammetry of different MnO_x_ nanomaterials in relation to their laccase-like reactivity. The results elucidate the rate behaviors of the laccase-like reactions of MnO_x_ materials under different conditions, and the role of oxygen and the electrochemical properties of the MnO_x_ materials. It appears that γ-MnO_2_ is the most potent to oxidize ABTS and E2 among the six different crystal structures, as well as to mediate O_2_ reduction, and thus provides the best catalytic performance. The findings help to further the understanding of the laccase-like reactivity of MnO_x_ materials, and provide a basis for future design and application of MnO_x_-based catalysts.

## Methods

### Chemicals

Laccase from *Pleurotus ostreatus* (EC 420-150-4), 2,2′-azinobis-(3-ethylbenzthiazoline-6-sulfonate) (ABTS) and 17β-Estradiol (E2, >98% purity) were purchased from Sigma-Aldrich (St. Louis, MO). The buffer salts acetic acid were ACS grade and from Fisher Scientific (Pittsburgh, PA). HPLC-grade acetonitrile (ACN) and methanol were also obtained from Fisher Scientific (Pittsburgh, PA). Chemical reagents of analytical grade including KMnO_4_, MnSO_4_·H_2_O, MnCl_2_, H_2_SO_4_, glucose, and hydrazine were purchased from Sigma-Aldrich (St. Louis, MO) and used as received.

### Preparation of MnO_x_ nanomaterials

The nanoparticles of *α*-, *β*-, *γ*- and *δ*-crystalline MnO_2_ and Mn_3_O_4_ were prepared by typical hydrothermal methods according to previously reported procedures^9, 42^, and *ε*-MnO_2_ nanorods (product ID 8005NJ, diameters 5–30 nm, lengths 80–100 nm, epsilon order) was provided by Nanostructured & Amorphous Materials, Inc. The synthesis procedures of all nanomaterials are described in detail in supplementary materials (Text S1), while a brief description of that for *α*-MnO_2_ is provided here as an example. MnSO_4_·H_2_O (0.845 g) and KMnO_4_ (1.94 g) were dissolved separately in 40 mL H_2_O and then combined in a Teflon-lined autoclave with stirring. The stirred solution was heated at 160 °C for 12 hours. The obtained suspension was centrifuged, decanted and washed with pure water to remove impurities until the pH value of the wastewater reached around 7.0, and the precipitate was then dried in an oven at 120 °C for 10 h in air. The morphology of these MnO_x_ nanomaterials was characterized by scanning electron microscopy (SEM) with images shown in Fig. S1. The phase of the MnO_x_ nanomaterials were identified by powder X-ray diffraction (XRD) using Cu Kα radiation with a step size of 0.05° and a sampling time of 2 s per step (Fig. S2). From the Fig. S2a and b, the X-ray diffraction patterns of MnO_x_ nanomaterials before and after oxidation reaction with E2 showed that the structure of six used manganese oxides is pretty much the same as the new ones. The Brunauer, Emmett, and Teller (BET) surface areas of the six nanomaterials were reported to be ranging from 32.9 to 193.9 m^2^/g (Table S1).

### Laccase-like reactions

A laccase activity assay method involving ABTS as the substrate^43, 44^ was used to evaluate the laccase-like reaction of MnO_x_. The reaction was conducted at room temperature in a 1-cm light path cuvette containing 3.4 mL of an ABTS solution in 0.1-M acetate buffer at pH 6.0. A MnO_x_ nanomaterial in 20-µL water slurry was added to the system at 30 μg/mL, and the reactor was hand shaken and then placed in a UV-Vis spectrophotometer (Beckman DU 800 spectrophotometer, Beckman Instruments Inc.) to record the absorbance change at 420 nm for five minutes. The initial reaction rate (*v*_initial_) was calculated as the rate of ABTS concentration change (ε_420_ = 36 000 M^−1^ cm^−1^)^45^ over the first 5 minutes. A series of experiments were performed with varying concentrations of ABTS (0.01, 0.1, 0.5, 1, 2, 5, and 10 mM) at pH 6.0, and with 2.0 mM ABTS at different pH (3.0, 4.0, 5.0, 6.0, 7.0 and 8.0) that was adjusted by 0.1 M HAc and 0.1 M NaAc.

The laccase-like reaction of MnO_x_ was also measured for longer term (up to 120 minutes) in 2.0-mM ABTS solution at pH 6.0 with oxygen saturated or depleted to evaluate the role of oxygen. The ABTS solution was sparged with pure O_2_ or N_2_ gas for at least 30 min prior to the experiment, and the dissolved oxygen concentration was measured as 21.5 and 0.30 mg/L, respectively, by a dissolved oxygen (DO) meter (YSI 5100, YSI Inc.). The reaction was conducted in a closed cuvette containing 3.4-mL ABTS solution and 0.1-mg MnO_x_ nanomaterial. After being hand shaken, the absorbance at 420 nm was recorded by the UV-Vis spectrophotometer for 120 minutes. The release of Mn(II) in solution was also determined in selected samples using the formaldoxime spectrophotometric method^19^ (see details in Text S2).

### Removal of 17-β estradiol in water by MnO_x_

Experiments to examine the removal of E2 in water by MnO_x_ nanomaterials were performed using 250-mL flasks as the reactors following a procedure that we have employed previously^38^. Each reactor contained 10-μM E2 in 100 mL 10-mM HAc/NaAc buffer with pH adjusted to 3.0, 4.0, 5.0, 6.0, or 7.0 by HAc and NaAc, and 1 mM of a MnO_x_ nanomaterial. The reaction was also performed in 10-mM acetate buffer at pH 4.0 containing 10-μM E2 and 2.0-μM MnO_x_ with O_2_ saturated (21.5 mg/L) or depleted (0.30 mg/L). The reactor was shaken at 180 r·min^−1^ under 25 °C, and 3 mL of the suspension was sampled at each prescribed sampling time, and transferred through a 0.2-μm polypropylene membrane (VWR International), followed by addition of 3-mL methanol to quench the reaction. One mL of the mixture was then sampled for HPLC analysis (the detail is provided in Text S3). All experiments were conducted in triplicate. Also, the intermediates and products of E2 reaction with manganese oxides were identified elaborately using a high resolution hybrid quadrupole time-of-flight mass spectrometer (Triple TOF 5600, AB Sciex, Foster City, CA) equipped with an electrospray ion source. The γ-MnO_2_ was selected as the representative nanomaterial for E2 removal, which the detail was shown in the Text S4 (HPLC-MS/MS analysis for E2) in supplementary materials.

### Electrochemical Characterization

Cyclic voltammetry (CV) and linear sweep voltammetry (LSV) were performed with a CHI6015D electrochemical workstation in a three-electrode electrochemical cell comprising a Pt wire counter electrode, a saturated Ag/AgCl reference electrode, and a glassy carbon electrode (diameter, 3 mm; CH Instruments, Inc.) as the working electrode at room temperature. The working electrodes were prepared according to previously reported procedures^46, 47^. In brief, the electrodes were firstly polished with 1.0, 0.5, and 0.05 μm alumina (CH Instruments, Inc.) in sequence to obtain a mirror finish. Six mg of a manganese oxide nanomaterial was dispersed in a mixture containing 0.5 mL water and 0.5 mL isopropyl alcohol by sonication for 30 minutes, followed by the addition of 20 μL Nafion solution (5 wt %, Sigma-Aldrich) and ultrasonicated for another 1 hour. Five μL of the sonicated solution was transferred onto the working electrode and air-dried before use. The effective surface area of the glassy carbon electrode was 0.1396 cm^2^, and thus the mass loading of the manganese oxide was about 0.21 mg/cm^2^ on the working electrode. The voltammetry was performed in 1.0 M KCl as the supporting electrolyte, which pH was adjusted to 3.0, 4.0, 5.0, 6.0, 7.0, or 8.0 using 0.1 M HAc and 0.1 M NaAc solution. The electrolyte was saturated with N_2_ or O_2_ for 30 min prior to each measurement. The voltammetry was collected at a potential scan rate of 0.02 V/s and the onset potential and peak potential were identified by the CHI6015D electrochemical software.

## Electronic supplementary material

Supplementary data
