# Supplementary material for: The laccase-like reactivity of manganese oxide nanomaterials for pollutant conversion: rate analysis and cyclic voltammetry
Source: Sci Rep. 2017 Aug 10;7:7756. doi: 10.1038/s41598-017-07913-2 (PMC5552746; doi:10.1038/s41598-017-07913-2)
Supplement: Supplementary file 1 — Supplementary data [file 41598_2017_7913_MOESM1_ESM.pdf]

Supplementary data

The laccase-like reactivity of manganese oxide nanomaterials for pollutant conversion: rate analysis and cyclic voltammetry

Xinghao Wang[a,b], Jiaoqin Liu[a], Ruijuan Qu[a*], Zunyao Wang[a], Qingguo Huang[b*]

[a] State Key Laboratory of Pollution Control and Resources Reuses, School of the Environment, Nanjing University, Nanjing 210023, P. R. China

[b] College of Agricultural and Environmental Sciences, Department of Crop and Soil Sciences, University of Georgia, Griffin, Georgia 30223, United States

*Correspondence and requests for materials should be addressed to R.Q. (email: quruijuan0404@nju.edu.cn) and Q.H. (email: qhuang@uga.edu)

Number of pages (including this page): 18

Number of Texts: 4

Number of Figures: 8

Number of Tables: 3

Text S1. Synthesis of MnO_x Materials

The five different crystalline MnO_x were prepared by typical hydrothermal methods according to previously reported procedures (Pokhrel et al., 2015).

1. Synthesis of α-MnO_2. $KMnO_4$ (1.94 g) and $MnSO_4$ (0.845 g) were dissolved separately in ten milliliter of H_2O and then combined in a Teflon-lined autoclave. The solution was heated at 160 ℃ for 12 hours. The resulting product was centrifuged, decanted and washed with H_2O to remove unwanted ions. The solid was dried in air.

2. Synthesis of β-MnO_2. In a typical procedure, $MnCl_2$ (0.475 g) and $KMnO_4$ (0.2528 g) were dissolved separately in 10 mL H_2O. The solutions were mixed together and stirred for 1 hour. The stirred solution was heated in a Teflon-lined autoclave at 180 ℃ for 48 hours. The resulting solid was washed with H_2O and dried at 180 ℃ for 18 hours.

3. Synthesis of γ-MnO_2. $MnSO_4$ H_2O (4.25 g) and $KMnO_4$ (1.715 g) were ground together until evenly mixed. The mixture was dissolved in 100 mL of H_2O with stirring and 50 mL of 1 M H_2SO_4 was added. The reaction mixture was stirred for 20 hours at room temperature. The solution was centrifuged, decanted and washed once with ethanol and twice with H_2O. The solid was dried at 70 ℃ overnight.

4. Synthesis of δ-MnO_2. A 1.4 M aqueous glucose solution was added to a 0.38 M aqueous $KMnO_4$ solution in a 2:5 ratio, forming a brown gel. Water was periodically drained over 2 hours as the gel underwent syneresis. The gel was then dried at 110 ℃ for 24 hours followed by calcination at 400 ℃ for 2 hours. The resulting powder was washed

with H_2O to remove impurities and dried at 90 ℃.

5. Synthesis of Mn_3O_4. An 80% hydrazine solution (5 mL) was added dropwise to an aqueous solution of 0.2 M $KMnO_4$ (20 mL), and the solution was stirred at room temperature for 12 hours. The resulting solid was centrifuged, decanted, washed with H_2O, and dried at 110 ℃ for 10 hours.

Text S2. Mn(II) determination

The formaldoxime spectrophotometric method (Morgan, 2005) was used to determine concentrations of Mn^{2+}(aq). The study followed a modified procedure as follows: Two mL of the sample suspension was prior pipetted and filtered using the microfilter to remove the manganese oxide particles. Then one mL of filtrate placed into a 15 mL tube for Mn(II) determination. The volume of the tube was adjusted to 6 mL, and the following reagents were added: 1mL of 1 M EDTA solution, 1.2 mL of formaldoxime reagent (stock prepared by dissolving hydroxylamine hydrochloride in MilliQ doubly-distilled water, adding 37% formaldehyde solution, and making to volume), 3 mL of 5 M NaOH, and MilliQ water for a final volume of 15 mL. Standards of 0.1, 0.2, 0.4, 0.6, 1.0, and 3.0 ppm Mn are prepared by dilution from a 1000 ppm stock Mn(II) solution. Blanks, standards, and samples are analyzed for absorbance at 450 nm in a Beckman DU 800 spectrophotometer.

Text S3. HPLC analysis for E2

E2 concentration was quantified using a HPLC (Shimadzu LC 20TA) equipped with a C18 column (25 cm × 4.6 mm, 5 μm particle, Supelco) and a diode array detector (SPD-M20A) at 40 ℃. The flow rate was set at 0.8 mL $\cdot$ min^{-1}, the injection volume was 20 μL, and the gradient was programmed as following: 20 min, 40% ACN; 30 min, 100% ACN; 31 min, 100% ACN; 35 min, 40% ACN.

Text S4. HPLC-MS/MS analysis for E2

The intermediates and products of E2 reaction with manganese oxides were identified elaborately using a high resolution hybrid quadrupole time-of-flight mass spectrometer (Triple TOF 5600, AB Sciex, Foster City, CA) equipped with an electrospray ion source. The γ-MnO_2 was selected as the representative nanomaterial for E2 removal in the additional experiment. The samples were taken from the reaction system initially containing 10.0 μM E2 and 1mM γ-MnO_2 in 100 mL acetate buffer (pH 4.0, 10 mM) with pH 4.0 after 0.5, 1, 2, 4, and 6 h of reaction, and control samples that didn't contain γ-MnO_2. The HPLC-MS condition are provided below in detail. A 5 μL sample was injected into the source using an Agilent 1260 infinity high performance liquid chromatography (HPLC) system. Chromatographic separation was performed on a Thermo BDS Hypersil C_{18} column (2.1 mm × 100 mm, particle size: 2.4 μm; Thermo Fisher Scientific, Waltham, MA) maintained at 30 °C, and the mobile phase consisting of 0.3% formic acid in water (A) and methanol (B) was eluted at a flow rate of 0.2 mL/min. The linear gradient was decreased from 90 % A (2 min) to 10 % A in 1 min, maintained for 23 min, then returned to the starting condition in 1 min, followed by an 8 min equilibration. Mass spectra (m/z 70-1000) were recorded in negative ion mode by using an electrospray ion (ESI) source with the following ion source parameters: ion source gas 1, -55 psi; ion source gas 2, -55 psi; curtain gas, -35 psi; temperature, 550 °C; ionspray voltage floating, -4500 V; declustering potential, -80 V; collision energy, -10 V. Nitrogen

gas was used throughout. Following the MS analysis, the individual parent ions of the possible transformation products of E2 were manually selected for MS/MS analysis. A sweeping collision energy setting of -30 ± 10 V was applied for collision-induced dissociation. The high-resolution LC-MS data were acquired with Analyst TF software (Version 1.6, AB Sciex) and processed using PeakView software (Version 1.2, AB Sciex).

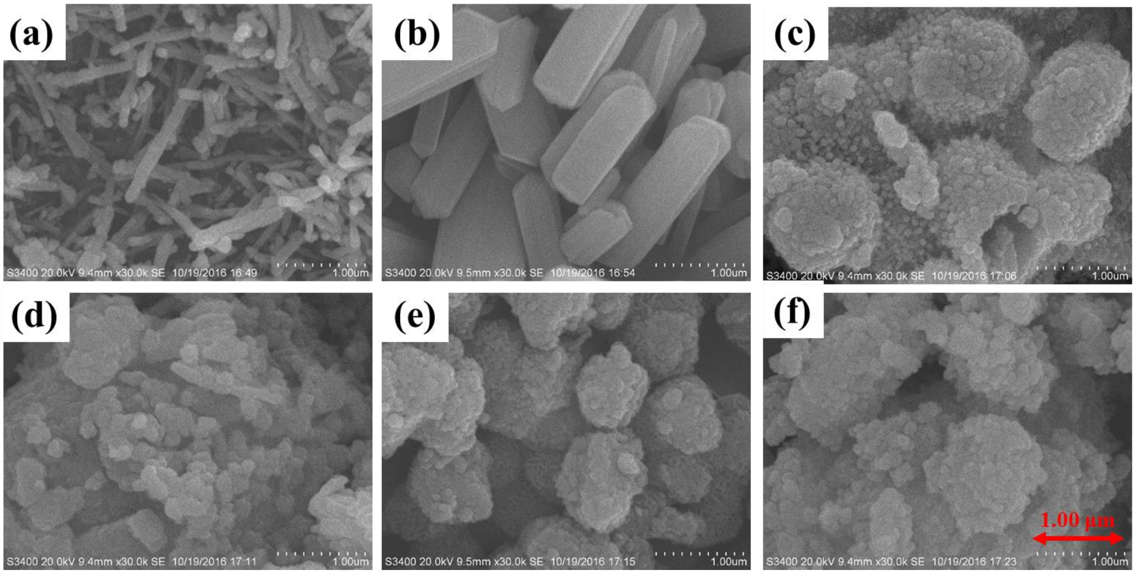

Fig. S1. FE-SEM images of MnO_x nanomaterials (a: α-MnO_2; b: β-MnO_2; c: γ-MnO_2; d: δ-MnO_2; e: ε-MnO_2; and f: Mn_3O_4) (bar 1.00 μm).

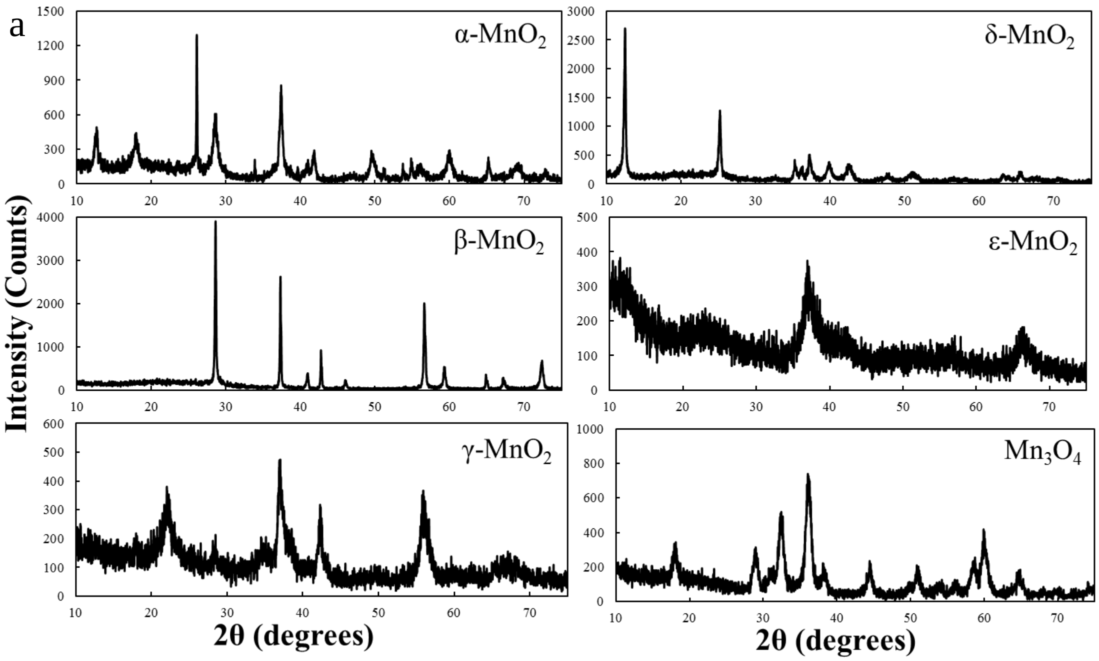

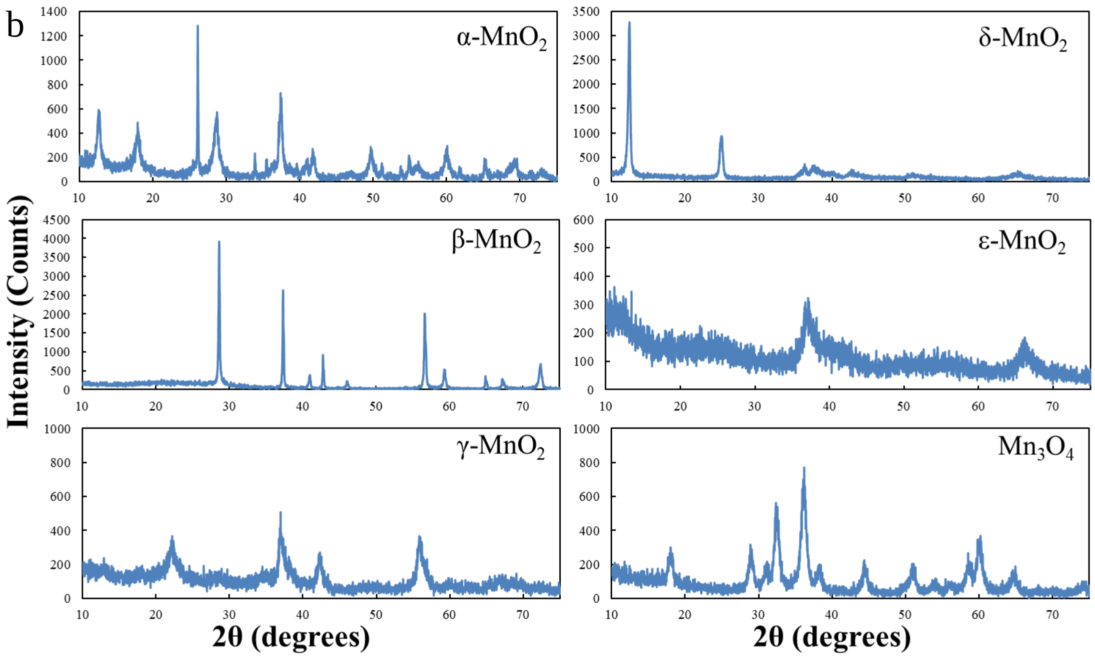

Fig. S2. Powder X-ray diffraction patterns of six MnO_x nanomaterials (a) before and (b) after oxidation with E2.

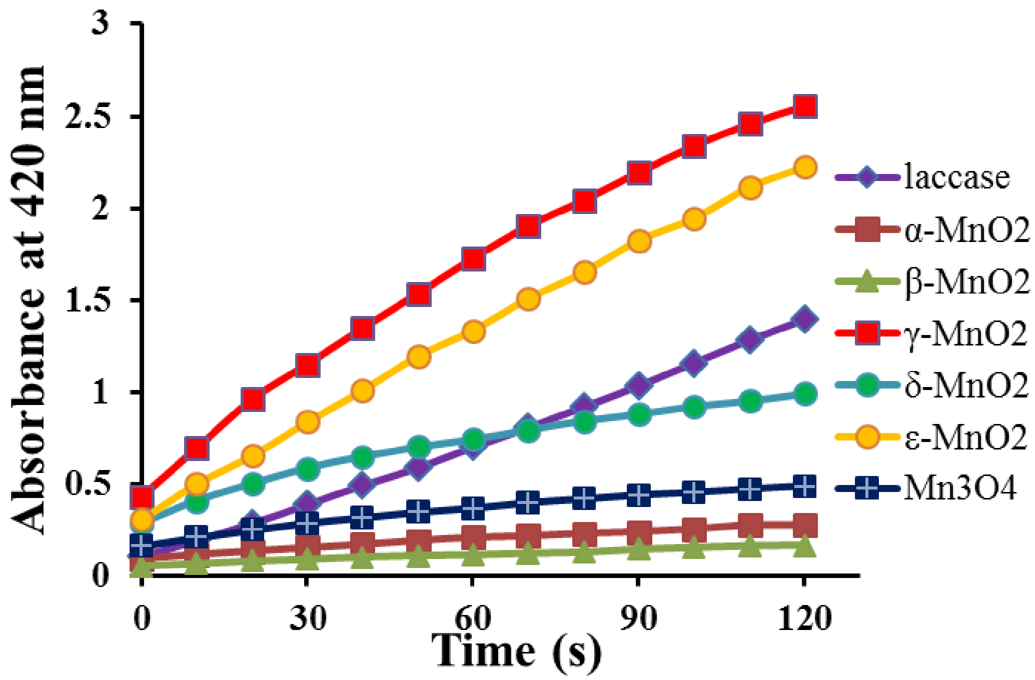

Fig. S3. The oxidation of ABTS (2 mM) by laccase (5 mg/mL) and different MnO_x (5 mg/mL) at pH 6.0. The result shows that the reaction rates of γ-MnO_2 (the red line) and ε-MnO_2 (the yellow line) are greater than the natural laccase (the purple line).

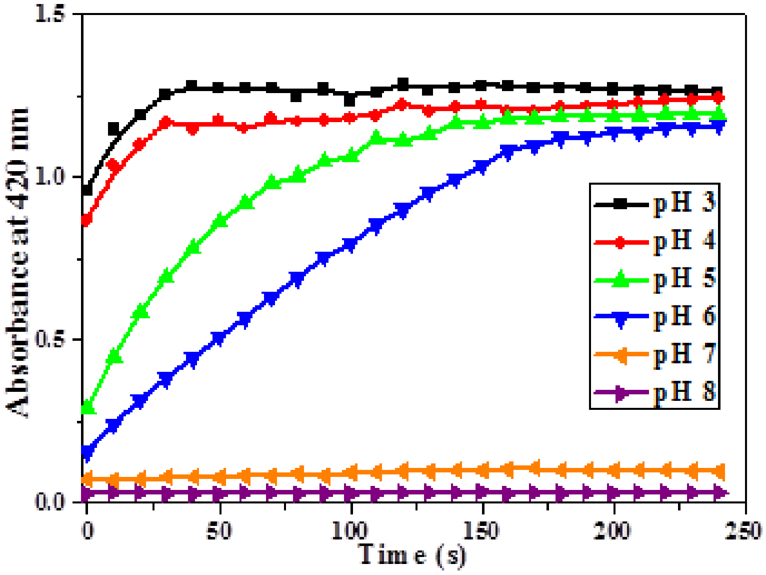

Fig. S4. The oxidation of ABTS (2 mM) by γ-MnO_2 (336 μM) under different pH. The initial reaction rate constant ($k_{initial}$) of ABTS oxidation at different pH is obtained by fitting the data to the pseudo-first-order rate equation $\ln (C_0/C_t) = k_{initial}$ t, where C_0 is the initial concentration of ABTS, and C_t is the residual concentration of ABTS at reaction time t.

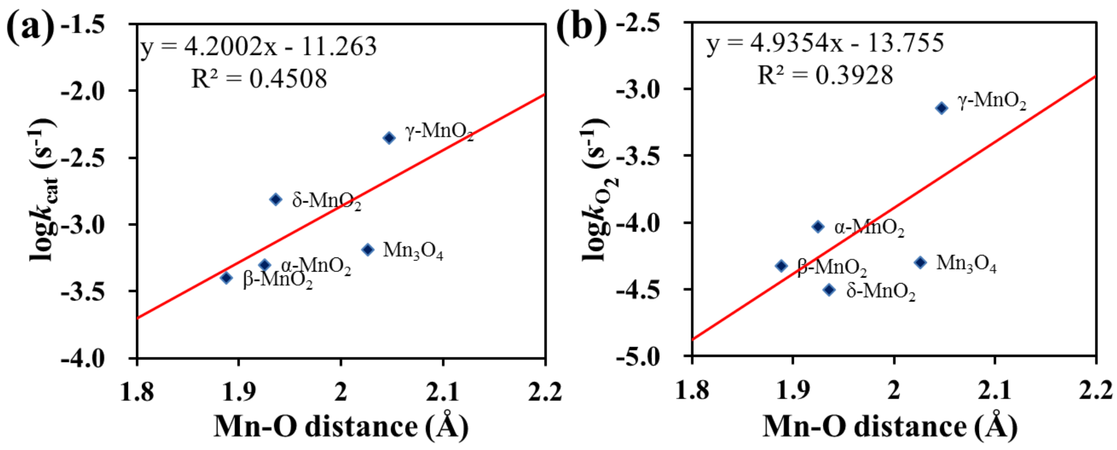

Fig. S5. (a) The relationship between logk_{cat} of ABTS initial reaction and the Mn-O bond length in different MnO_x nanomaterials. (b) The relationship between logk_{O2} of ABTS oxidation and the Mn-O bond length of different MnO_x nanomaterials.

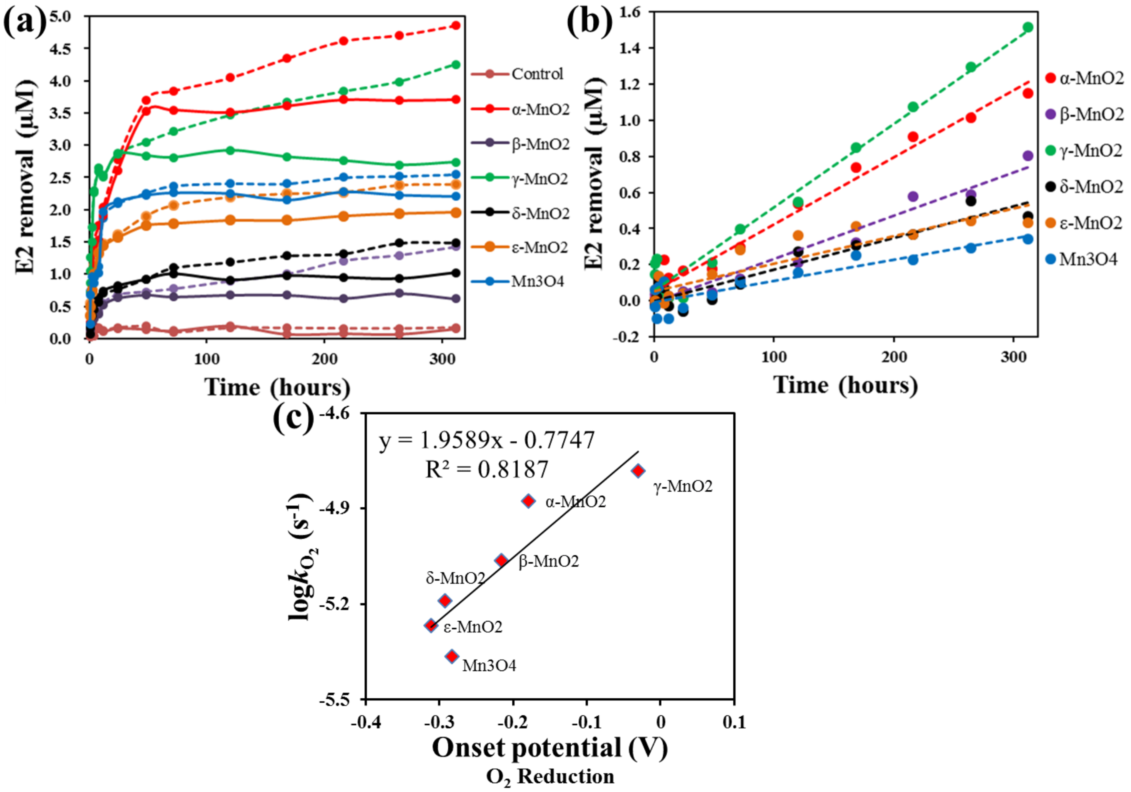

Fig. S6. (a) E2 removal by different MnO_x in systems with O_2 saturated (21.5 mg/L, dash line) or depleted (0.30 mg/L, solid line). Initial conditions: [E2] = 10 μM, [MnO_x] = 2.0 μM, pH 4.0. (b) The difference of E2 removal by MnO_x between the two systems tested in a. (c) The correlation between the $\log k_{O2}$ and the onset potential of O_2 reduction by different MnO_x nanomaterials at pH 4.0.

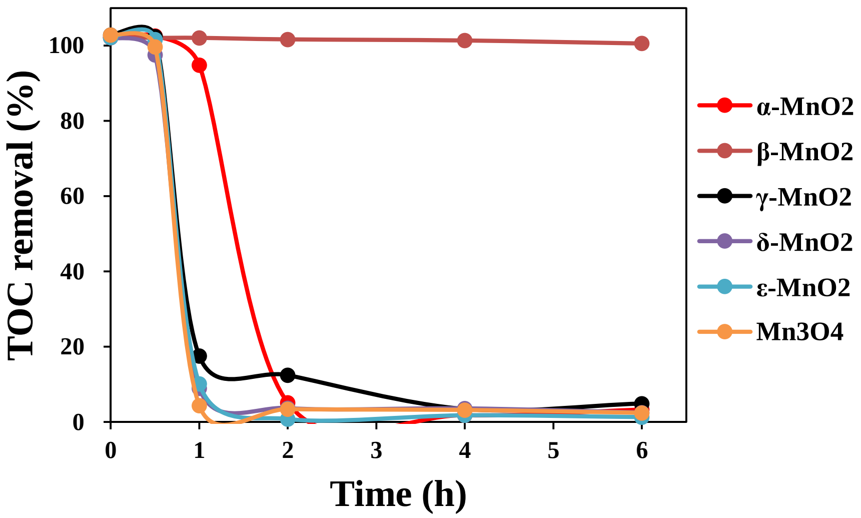

Fig. S7. Removal percentage rate of the TOC vs. reaction time in solution with $E2_o$=10 μM in the presence of six different manganese oxides with 1.0 mM.

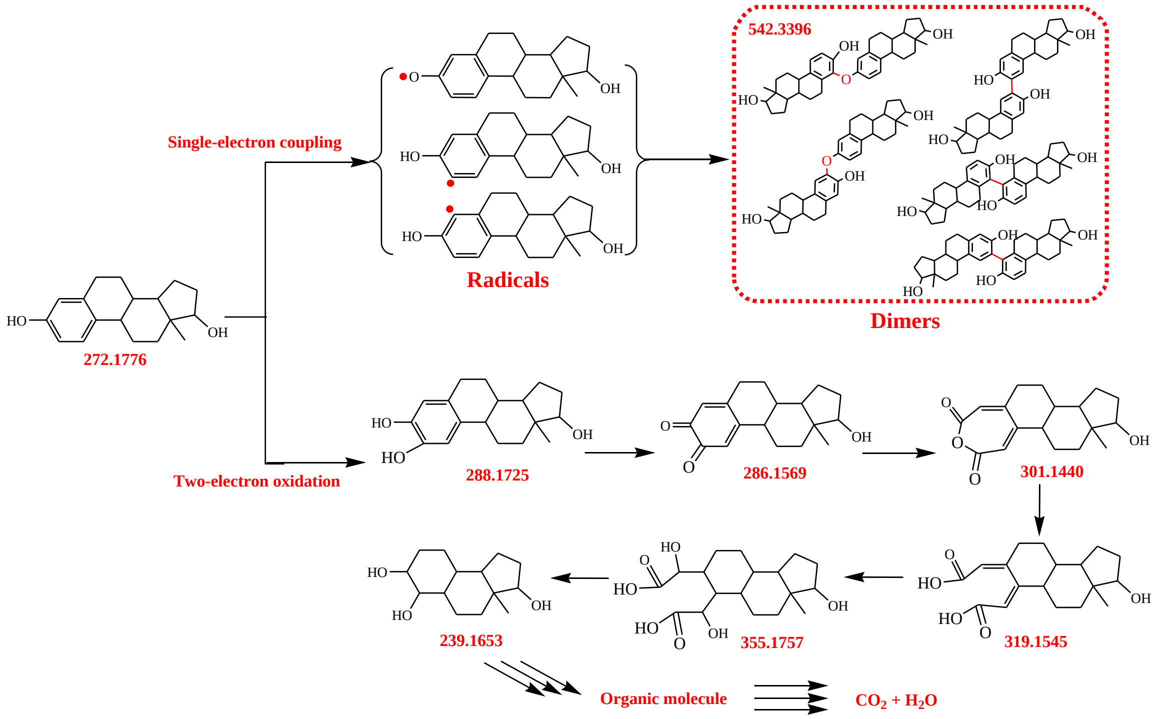

Fig. S8. The product intermediates and two pathways of E2 conversion in the presence of γ-MnO_2.

Table S1. Manganese oxides nanomaterials obtained under different reaction conditions.

Sample	Phase	Experimental conditions			Surface area (m^2/g)
		oxidant	reductant	T (°C)	
1	α-MnO_2	$KMnO_4$	$MnSO_4$ H_2O	160	34.4
2	β-MnO_2	$KMnO_4$	$MnCl_2$	180	56.5
3	γ-MnO_2	$KMnO_4$ and H_2SO_4	$MnSO_4$ H_2O	25	167.4
4	δ-MnO_2	$KMnO_4$	glucose	400	32.9
5	ε-MnO_2	no	$Mn(NO_3)_2$ $4H_2O$	550	193.9
6	Mn_3O_4	$KMnO_4$	80% hydrazine	25	49.3

Table S2. Rate constants for the ABTS oxidation by γ-MnO_2 under different pH values.

pH	3.0	4.0	5.0	6.0	7.0	8.0
$k_{initial}$ (s^{-1})	1.42×10^{-3}	1.57×10^{-3}	1.11×10^{-3}	5.50×10^{-4}	1.72×10^{-5}	2.66×10^{-6}

Table S3. The average Mn-O distance in selected structures of manganese oxides.

MnO_x	Avg. Mn-O distance, Å	References
α-MnO_2	1.925	(Robinson et al., 2013)
β-MnO_2	1.888	(Robinson et al., 2013)
γ-MnO_2	2.047	(Kohler et al., 1997)
δ-MnO_2	1.936	(Robinson et al., 2013)
ε-MnO_2	-	-
Mn_3O_4	2.026	(Robinson et al., 2013)

References:

Kohler, T., Armbruster, T., Libowitzky, E., 1997. Hydrogen bonding and Jahn–Teller distortion in groutite, α-MnOOH, and manganite, γ-MnOOH, and their relations to the manganese dioxides ramsdellite and pyrolusite. J. Solid State Chem. 133 (2), 486-500.

Morgan, J.J., 2005. Kinetics of reaction between O_2 and Mn(II) species in aqueous solutions. Geochim. Cosmochim. Acta 69 (1), 35-48.

Pokhrel, R., Goetz, M.K., Shaner, S.E., Wu, X., Stahl, S.S., 2015. The “best catalyst” for water oxidation depends on the oxidation method employed: a case study of manganese oxides. J. Am. Chem. Soc. 137 (26), 8384-8387.

Robinson, D.M., Go, Y.B., Mui, M., Gardner, G., Zhang, Z., Mastrogiovanni, D., Garfunkel, E., Li, J., Greenblatt, M., Dismukes, G.C., 2013. Photochemical water oxidation by crystalline polymorphs of manganese oxides: structural requirements for catalysis. J. Am. Chem. Soc. 135 (9), 3494-3501.
